# *Porphyromonas gingivalis*-Lipopolysaccharide Induced Caspase-4 Dependent Noncanonical Inflammasome Activation Drives Alzheimer’s Disease Pathologies

**DOI:** 10.3390/cells14110804

**Published:** 2025-05-30

**Authors:** Ambika Verma, Gohar Azhar, Pankaj Patyal, Xiaomin Zhang, Jeanne Y. Wei

**Affiliations:** Donald W. Reynolds Department of Geriatrics and Institute on Aging, University of Arkansas for Medical Sciences, Little Rock, AR 72205, USA; averma@uams.edu (A.V.); azhargohar@uams.edu (G.A.); ppatyal@uams.edu (P.P.); zhangxiaomin@uams.edu (X.Z.)

**Keywords:** *Porphyromonas gingivalis*, lipopolysaccharide, caspase-4, neuroinflammation, oxidative stress, mitochondrial dysfunction

## Abstract

Chronic periodontitis, driven by the keystone pathogen *Porphyromonas gingivalis*, has been increasingly associated with Alzheimer’s disease (AD) and AD-related dementias (ADRDs). However, the mechanisms through which *P. gingivalis*-lipopolysaccharide (LPS)-induced release of neuroinflammatory proteins contribute to the pathogenesis of AD and ADRD remain inadequately understood. Caspase-4, a critical mediator of neuroinflammation, plays a pivotal role in these processes following exposure to *P. gingivalis*-LPS. In this study, we investigated the mechanistic role of caspase-4 in *P. gingivalis*-LPS-induced IL-1β production, neuroinflammation, oxidative stress, and mitochondrial alterations in human neuronal and microglial cell lines. Silencing of caspase-4 significantly attenuated IL-1β secretion by inhibiting the activation of the caspase-4-NLRP3-caspase-1-gasdermin D inflammasome pathway, confirming its role in neuroinflammation. Moreover, caspase-4 silencing reduced the activation of amyloid precursor protein and presenilin-1, as well as the secretion of amyloid-β peptides, suggesting a role for caspase-4 in amyloidogenesis. Caspase-4 inhibition also restored the expression of key neuroinflammatory markers, such as total tau, VEGF, TGF, and IL-6, highlighting its central role in regulating neuroinflammatory processes. Furthermore, caspase-4 modulated oxidative stress by regulating reactive oxygen species production and reducing oxidative stress markers like inducible nitric oxide synthase and 4-hydroxynonenal. Additionally, caspase-4 influenced mitochondrial membrane potential, mitochondrial biogenesis, fission, fusion, mitochondrial respiration, and ATP production, all of which were impaired by *P. gingivalis*-LPS but restored with caspase-4 inhibition. These findings provide novel insights into the role of caspase-4 in *P. gingivalis*-LPS-induced neuroinflammation, oxidative stress, and mitochondrial dysfunction, demonstrating caspase-4 as a potential therapeutic target for neurodegenerative conditions associated with AD and related dementias.

## 1. Introduction

During aging, the immune system becomes less effective, making older adults more vulnerable to infections like periodontal disease, where bacterial infections cause plaque buildup and gum inflammation, resulting in gingivitis [1,2]. *Porphyromonas gingivalis* (*P. gingivalis*), a Gram-negative rod-shaped bacterium, is the primary pathogen in chronic periodontitis and is also considered a potential contributor to Alzheimer’s disease (AD) and AD-related dementias (ADRDs) [3,4,5]. A recent bidirectional Mendelian randomization study suggested a positive association between *P. gingivalis* infection and the development of AD [6]. *P. gingivalis* releases various virulence factors, including lipopolysaccharide (LPS), outer membrane vesicles (OMVs), and gingipains, which trigger systemic inflammatory events responsible for neuroinflammation associated with AD and ADRD [7,8]. Recent studies have detected *P. gingivalis* and its virulence factors in the brains of patients with AD and have linked *P. gingivalis*-LPS to neuroinflammation and neurodegeneration [9,10,11].

LPS, a key component of the *P. gingivalis* outer membrane, reaches neuronal cells through OMVs, which deliver LPS into the cytosol via direct membrane fusion or endocytosis [12,13]. *P. gingivalis*-LPS is also internalized via receptor-mediated endocytosis, where it engages with pattern recognition receptors, including Toll-like receptor 4 [14]. Human caspase-4 and mouse caspase-11 are homologous proteins that are activated when extracellular LPS is internalized by host cells [15]. Caspase-4 directly senses and binds cytosolic LPS, promoting its oligomerization and activation, leading to the activation of the noncanonical inflammasome [16]. Activated caspase-4 cleaves gasdermin D (GSDMD), generating the N-terminal fragment GSDMD-N, which inserts into the plasma membrane to form pores and trigger pyroptosis, a form of programmed cell death [17]. These GSDMD-N-mediated pores also facilitate K^+^ efflux, which is sensed by the NLRP3 inflammasome, leading to the activation of caspase-1. Activated caspase-1 further promotes the maturation and secretion of IL-1β and contributes to pyroptosis [18,19]. The caspase-4-dependent noncanonical inflammasome activation plays a pivotal role in driving inflammation, promoting the production of reactive oxygen species (ROS), which can also activate a series of events that may lead to the hyperactivation of NLRP3, resulting in IL-1β production [20]. Recent studies highlight the functional interaction between human caspase-4 and NLRP3 to induce caspase-1-mediated inflammatory responses [21,22,23]. In periodontal pathogenesis, *P. gingivalis* bacteria can trigger inflammation by activating NLRP3-dependent secretion of IL-1β [19]. However, the role of caspase-4 in *P. gingivalis*-LPS-mediated AD-related neuropathology remains unexplored.

The progression of amyloid and tau pathologies is a hallmark of AD, and neuroinflammation driven by microbial components like *P. gingivalis*-LPS has been shown to exacerbate these pathologies [24,25,26,27]. Previous studies have shown that *P. gingivalis* or its LPS induce amyloidogenic processing of amyloid precursor protein (APP), leading to the production of amyloid-β (Aβ), which forms plaques in the brain [28,29,30,31]. Aβ is produced from APP using enzymes β-secretase and γ-secretase in the amyloidogenic pathway of APP processing. Presenilin-1 (PS1), a component of the γ-secretase complex, plays a critical role in the amyloidogenic processing of APP that leads to the production of amyloid-β_1–42_ [32]. Additionally, in AD models of APP and PS1, another crucial protein, reelin, has been found to co-localize with amyloid plaques [33,34]. It has been demonstrated that reelin links Aβ and tubulin-associated unit (tau), which are key proteins that have been implicated in AD pathogenesis [35]. This interaction between APP, PS1, reelin, Aβ, and tau may play a role in neurodegeneration and AD progression [32,33,34,35].

Neurodegeneration and AD pathology are also marked by various other hallmarks that disrupt neuronal function by causing oxidative stress and ROS production and impair energy supply by altering mitochondrial function [36]. Mitochondria are dynamic organelles that undergo continuous fission and fusion, regulated by GTPases [37,38,39]. Mitochondria are the primary source of adenosine triphosphate (ATP) through oxidative phosphorylation (OXPHOS), regulated by five multienzyme complexes (I–V) of the mitochondrial inner membrane, where complexes I and II have been identified as primary ROS producers [40,41]. Dysregulation of mitochondrial function leads to excessive ROS production and impaired ATP synthesis, both of which exacerbate oxidative stress that induces neuroinflammation and neurodegeneration [42,43,44]. In addition to their well-known role in energy production, mitochondria serve as key regulators of several non-energetic cellular processes and are central to the intrinsic pathway of apoptosis [45]. During *P. gingivalis* infection, mitochondrial involvement in apoptotic regulation has been observed in various host cell types, potentially contributing to tissue destruction and immune evasion [46]. Furthermore, mitochondria participate in transcellular signaling by generating ROS and releasing mitochondrial DNA (mtDNA), both of which can act as signaling molecules during infection [46]. *P. gingivalis* has been shown to modulate mitochondrial ROS production, thereby influencing inflammatory signaling cascades [47]. It has been reported that mitochondrial quality gets compromised by *P. gingivalis* or its LPS due to a range of challenges, such as inflammation and oxidative stress that may contribute to chronic inflammation and immune modulation observed in periodontitis [47]. Interestingly, *P. gingivalis* or its LPS-mediated ROS production and increased oxidative stress have also been reported in periodontal ligament fibroblasts, human neuroblastoma, and brain endothelial cells [48,49,50]. Recent studies have demonstrated that LPS from pathogenic enterohemorrhagic *Escherichia coli* activates caspase-4, GSDMD, and the NLRP3 inflammasome, which induces mitochondrial ROS production, IL-1β maturation, and pyroptosis [51]. However, it is well established that LPS molecules from different bacterial species—such as *P. gingivalis* and *E. coli*—differ markedly in their lipid A structure and immunological properties, resulting in divergent inflammasome activation profiles and cytokine responses [52]. Therefore, the specific molecular mechanisms underlying caspase-4 activation in response to *P. gingivalis* LPS, particularly in the context of mitochondrial dysfunction and neuroinflammation, remain to be fully elucidated.

Several studies have demonstrated that LPS-induced oxidative stress significantly enhances inducible nitric oxide synthase (iNOS) expression, leading to increased nitric oxide production and promoting inflammation and disease progression [53,54]. The overproduction of ROS can also damage lipids, resulting in lipid peroxidation and the generation of toxic products like 4-hydroxynonenal (4-HNE), which has been shown to be significantly elevated in AD [55,56]. Additionally, the aberrant production of ROS can also disrupt cellular function through manganese superoxide dismutase (MnSOD), an antioxidant enzyme that plays a key role in mitigating this damage [57].

We explored the mechanism by which *P. gingivalis*-LPS activates a caspase-4-dependent noncanonical inflammasome pathway, leading to the release of IL-1β and causing oxidative stress and mitochondrial dysfunction, which promotes neuroinflammation and potentially contributes to AD and ADRD. To our knowledge, this represents the first exploration of caspase-4-dependent noncanonical inflammasome activation in the framework of *P. gingivalis*-LPS-mediated neurodegenerative diseases. Overall, targeting the caspase-4 pathway may offer a promising therapeutic strategy to reduce *P. gingivalis*-LPS-induced neurotoxicity linked to AD and ADRD, and identifying specific molecular targets will be crucial in the development of future treatments aimed at alleviating these conditions.

## 2. Materials and Methods

### 2.1. Cell Culture and Treatments

Human SH-SY5Y neuroblastoma cells (ATCC CRL-2266) were cultured in Dulbecco’s Modified Eagle’s Medium/Nutrient Mixture F-12 (DMEM/F12) (ATCC, Cat# 11965092), and human HMC3 microglial cells (ATCC CRL-3304) were cultured in Minimum Essential Medium (MEM) (ATCC, Cat# 30-2003). Both cell lines were supplemented with 10% newborn bovine serum and 1% penicillin/streptomycin. Ultrapure *P. gingivalis*-LPS was sourced from InvivoGen (Cat# tlrl-ppglps) and Lipofectamine™ 2000 (LF2K) transfection reagent (Cat# 11668019) and OptiMEM cell growth media (Cat# 31985070) were obtained from Thermo Fisher Scientific (Waltham, MA, USA). Cells were transfected with *P. gingivalis*-LPS (10 μg/mL) in the presence of LF2K (1 μg/mL) in OptiMEM for 24 (SH-SY5Y cells) or 4 h (HMC3 cells) and incubated in a humidified incubator at 37 °C with 5% CO_2_ [31]. The selected concentration of 10 μg/mL for 24 h in SH-SY5Y cells or 4 h in HMC3 cells was based on findings from our previous studies [31,49]. In some experiments, cells were pretreated with 10 μM caspase-4 inhibitor Ac-LEVD-CHO (Cat# 27433) for 1 h before *P. gingivalis*-LPS transfection per the manufacturer’s instructions (Cayman Chemical).

### 2.2. Small Interfering RNA (siRNA) Mediated Knockdown Assay

SH-SY5Y and HMC3 cells were transfected with 20 nM of validated Silencer^®^ siRNA targeting human caspase-4 (AM51331, Assay ID 4036) and the Silencer™ Negative Control siRNA (AM4635) (Thermo Fisher Scientific, Waltham, MA, USA) using HiPerFect Transfection Reagent (Qiagen, Germantown, MD, USA), following the manufacturer’s protocol. Forty-eight hours post-transfection, SH-SY5Y cells were exposed to *P. gingivalis*-LPS for 24 h, while HMC3 cells were treated for 4 h, as described previously [31].

### 2.3. Enzyme-Linked Immunosorbent Assay (ELISA)

SH-SY5Y and HMC3 cells were plated at a density of 1.0 × 10^6^ cells per well in 6-well culture plates and transfected as described previously [31]. Non-transfected cells treated with 1 μg/mL LF2K were used as controls. After optimal incubation periods, the cell culture supernatants were collected and analyzed quantitatively for IL-1β, Aβ_1–40_, and Aβ_1–42_ using the human IL-1β Quantikine ELISA kit (Cat# DY201-05) and human Aβ (aa_1–40_ and aa_1–42_) Quantikine ELISA kits, (Cat# DAB142; DAB140B) following the manufacturer’s protocols (R&D Systems, Minneapolis, MN, USA). Phosphorylated tau (p-tau) at Threonine 181 and Threonine 217 was measured in the supernatants using p-tau Thr181 (Invitrogen, Cat# KHO0631, Waltham, MA, USA) and p-tau Thr217 (Cell Signaling Technology, Cat# 59672C, Danvers, MA, USA) ELISA kits, according to the manufacturer’s instructions.

### 2.4. Real-Time Quantitative Polymerase Chain Reaction

Total RNA was extracted using the RNeasy Mini Kit (Qiagen; Cat# 74104) combined with TRIzol^®^ reagent (Invitrogen, Cat# 15596026, Waltham, MA, USA) and RNA concentrations were determined using a Nanodrop ND-100 spectrophotometer (NanoDrop). cDNA synthesis was performed using the iScript™ cDNA Synthesis Kit (BioRad, Cat# 1708890, Hercules, CA, USA) according to the manufacturer’s instructions. Real-time quantitative PCR (RT-qPCR) was performed using PowerTrack™ SYBR Green Master Mix (ThermoFisher Scientific, Cat# A46012, Waltham, MA, USA), with gene expression changes analyzed via the 2^−ΔΔCT^ method, using 5S rRNA as the internal control. Relative expression levels were calculated by normalizing the expression change to the control for each gene as described previously [58]. The primer sequences for quantifying gene expression are described in Supplementary Table S1.

### 2.5. Western Blot Analysis

SH-SY5Y and HMC3 cells were transfected according to previously described protocols [31]. After optimal incubation periods, the cells were washed with ice-cold PBS and lysed using RIPA lysis buffer (Santa Cruz; Cat# sc-24948A, Dallas, TX, USA). The protein concentrations in the lysates were determined using the Pierce BCA Protein Assay Kit (Thermo Fisher Scientific; Cat# 23227, Waltham, MA, USA). For Western blot analysis, 50 µg samples of total protein were separated via sodium dodecyl sulfate-polyacrylamide gel electrophoresis (SDS-PAGE) and subsequently transferred to nitrocellulose membranes. The membranes were blocked with 5% non-fat dry milk in Tris-buffered saline with Tween-20 (TBST) for 1 h at room temperature, washed three times with TBST (10 min each), and incubated overnight at 4 °C with primary antibodies. The primary antibodies used included caspase-4 (1:500, Cat# 4450S), NLRP3 (1:1000, Cat# 13158S), caspase-1 (1:1000, Cat# 2225S), Gasdermin D (1:1000, Cat# 97558S), IL-1β (1:1000, Cat# 12242S), APP (1:1000, Cat# 2452S), Presenilin 1 (1:1000, Cat# sc-365450), reelin (1:1000, Cat# ab139691), PGC-1α (1:1000, Cat# sc-518025), PGC-1β (1:1000, Cat# sc-373771), Nrf1 (1:1000, Cat# sc-515360), mtTFA (1:1000, Cat# sc-166965), DRP1 (1:1000, Cat# sc-271583), Mfn1/Mitofusin 1 (1:1000, Cat# sc-166644), Mfn2/Mitofusin 2 (1:1000, Cat# sc-515647), OPA1 (1:1000, Cat# sc-393296), OXPHOS (1:5000, Thermo Fisher Scientific, Cat# 45-8099, Waltham, MA, USA), NOS2/iNOS Antibody (1:1000, Cat# sc-7271), HNE (1:1000, Cat# ab46545), SOD-2 (1:1000, Cat# sc-30080), and β-Actin (Cat# sc-47778). The following day, the membranes were washed three times with TBST (10 min each) and incubated with secondary antibodies for 1 h at room temperature. The secondary antibodies, anti-mouse HRP (Invitrogen, Cat# 62-6520, Waltham, MA, USA) and anti-rabbit AP (Bio-Rad, Cat# 64251130, Hercules, CA, USA), were diluted 1:5000 in blocking solution. After incubation, the membranes were washed three times with TBST (10 min each). Immunoreactive bands were visualized using the SuperSignal West Dura Luminol/Enhancer Solution (Thermo Fisher Scientific, Waltham, MA, USA, Cat# 1856145). Imaging was performed using the iBright™ CL1500 (Invitrogen, Waltham, MA, USA), and densitometric analysis of the bands was performed using ImageJ software v1.53t (National Institutes of Health, Bethesda, MD, USA).

### 2.6. Flow Cytometry Analysis

Mitochondrial reactive oxygen species (mtROS) levels were measured using MitoSOX Red (Thermo Fisher, Cat# M36008) as previously described [49]. SH-SY5Y cells were seeded at a density of 1 × 10^6^ cells per well and pretreated for 1 h with the caspase-4 inhibitor Ac-LEVD-CHO (10 μM), before *P. gingivalis*-LPS transfection. After 24 h of incubation, cells were stained with 5 µM MitoSOX Red for 10 min to measure mtROS production. To assess mitochondrial membrane potential (MMP), another similar set of experimental cells was stained with 5 µg/mL JC-1 fluorescent dye for 10 min (Thermo Fisher, Cat# T3168, Waltham, MA, USA). After staining, the cells were centrifuged and gently washed three times with warm HBSS buffer, after which flow cytometry analysis was performed. Fluorescence intensity was quantified using a flow cytometer (BD LSRFortessa™, Franklin Lakes, NJ, USA) and analyzed with FlowJo v10.8.1 software.

### 2.7. High-Resolution Respiratory Analysis

The Oxygraph-O2k high-resolution respirometer (Oroboros Instruments, Innsbruck, Austria) was used to examine the mitochondrial function of individual respiratory chain complexes (I-IV) in the permeabilized cells using digitonin (Sigma; Cat# D5628, St. Louis, MO, USA) as previously described [49]. Briefly, SH-SY5Y cells were pretreated for 1 h with the caspase-4 inhibitor Ac-LEVD-CHO (10 μM) before *P. gingivalis*-LPS transfection. After 24 h of incubation, cells were harvested, and 5 × 10^6^ cells each from the control and treatment group were incubated for 20 min at 4 °C with digitonin (8 μM/million cells) prepared in MiRO5 buffer to permeabilize the cells. The mitochondrial respiratory activity of different complexes was analyzed using a substrate–uncoupler–inhibitor–titration protocol as described previously [59]. Data were exported and analyzed with DatLab 6.2 software (Oroboros Instruments, Innsbruck, Austria), and cellular respiration of individual mitochondrial complexes was expressed as oxygen flux (pmol/s*Million Cells).

### 2.8. Real-Time Metabolic Flux Assays

The oxygen consumption rate (OCR), extracellular acidification rate (ECAR), and ATP rate assays were performed using an Agilent XFe96 Analyzer (Seahorse Bioscience, Agilent, Santa Clara, CA, USA), following previously established protocols and reagents [59]. Briefly, SH-SY5Y cells were seeded at a density of 5 × 10^4^ cells per well in XFe 96-well plates (Seahorse Bioscience, Agilent, Santa Clara, CA, USA), with complete media and incubated at 37 °C, 5% CO_2_, and 100% humidity. The cells were transfected with 20 nM of validated Silencer^®^ siRNAs targeting caspase-4 and a negative control using the HiPerFect Transfection Reagent (Qiagen; Cat# 301704, Germantown, MD, USA) according to the manufacturer’s instructions. After 48 h, the cells were transfected with 10 μg/mL *P. gingivalis*-LPS for 24 h, as previously described. Following treatment, cells were washed with Seahorse XF DMEM media (pH 7.4) and incubated for 1 h in the same media at 37 °C in a non-CO_2_ incubator. Real-time cell metabolic function was assessed using the Seahorse XFe96 Analyzer and the XF assay kits, reagents, and cell assay media, including the XF Cell Mito Stress Test Kit (Agilent; Cat# 103015-100, Santa Clara, CA, USA), XF Glycolytic Rate Assay Kit (Agilent; Cat# 103344-100, Santa Clara, CA, USA), and XF Real-Time ATP Rate Assay Kit (Agilent; Cat# 103592-100, Santa Clara, CA, USA),), as described previously [60]. OCR, ECAR, and ATP assay data were normalized to the equal number of cells in each condition, which was determined by performing a cell count using the trypan blue exclusion method [61].

### 2.9. Statistical Analysis

Experiments were conducted a minimum of three times, and the data are presented as means ± SEM, with *n* representing the number of experiments unless otherwise specified. One-way or two-way ANOVA followed by Tukey’s multiple comparisons test was performed for comparisons among multiple groups. A *p*-value of less than 0.05 was considered statistically significant. Statistical analyses were performed using Prism 10.0 (GraphPad Software).

## 3. Results

### 3.1. Silencing of Caspase-4 Reduces P. gingivalis-LPS-Induced Secretion of IL-1β

**Figure 1:**
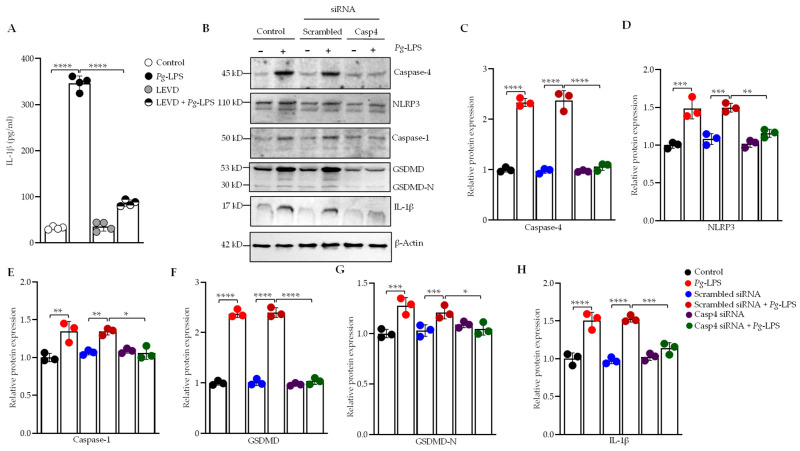
*P. gingivalis*-LPS induces IL-1β secretion via a caspase-4 dependent non-canonical inflammasome pathway. SH-SY5Y cells were pretreated with Ac-LEVD-CHO (a caspase-4 inhibitor) for 1 h before LPS treatment. (**A**) After 24 h, culture supernatants were collected, and IL-1β secretion was quantified by ELISA (*n* = 4). (**B**) Representative Western blots depict the upregulation of caspase-4, NLRP3, caspase-1, GSDMD, GSDMD-N, and IL-1β in response to *P. gingivalis*-LPS, along with the reversal of this effect following caspase-4 silencing via siRNA. β-Actin was used as a loading control. (**C**–**H**) Quantification of relative protein expression of caspase-4, NLRP3, caspase-1, GSDMD, GSDMD-N, and IL-1β, normalized to β-Actin, is presented in the graphs (*n* = 3). Data are expressed as mean ± SEM and represent at least three independent experiments. Statistical significance is indicated as follows: * *p* < 0.05; ** *p* < 0.01; *** *p* < 0.001; **** *p* < 0.0001, determined by one-way ANOVA with Tukey’s multiple comparisons test.

**Figure 2:**
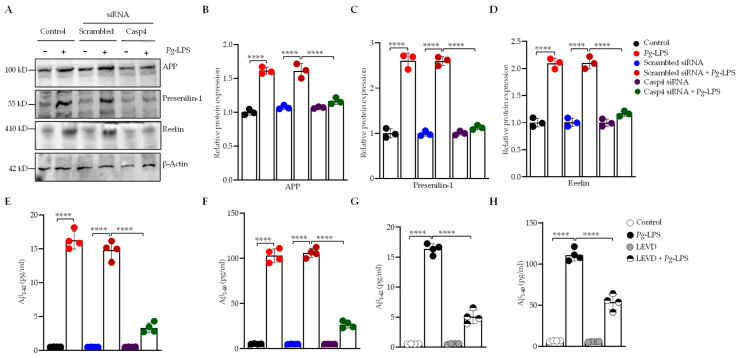
*P. gingivalis*-LPS upregulates Alzheimer’s disease-associated proteins APP, PS1, reelin, and Aβ in a caspase-4 dependent manner in SH-SY5Y cells. (**A**) Representative Western blots demonstrate that silencing of caspase-4 using siRNA restores the protein expression of APP, PS1, and reelin following *P. gingivalis*-LPS treatment in SH-SY5Y cells (*n* = 3). β-Actin was used as a loading control. (**B**–**D**) Quantification of relative protein levels of APP, PS1, and reelin normalized to β-Actin is shown in the graphs. (**E**,**F**) ELISA analysis of Aβ_1–42_ and Aβ_1–40_ peptides in SH-SY5Y cells following *P. gingivalis*-LPS treatment, in combination with caspase-4 siRNA (*n* = 4). (**G**,**H**) ELISA analysis of Aβ_1–42_ and Aβ_1–40_ peptides following *P. gingivalis*-LPS treatment and caspase-4 inhibition with Ac-LEVD-CHO (*n* = 4). Data are expressed as mean ± SEM and represent at least three independent experiments. Statistical significance is indicated as follows: **** *p* < 0.0001 as determined by one-way ANOVA with Tukey’s multiple comparisons test.

Caspase-4, a cytosolic sensor involved in detecting intracellular LPS, was investigated to assess its role in the *P. gingivalis*-LPS-mediated activation of the caspase-4-dependent signaling pathway in SH-SY5Y and HMC3 cells. Additionally, the production of IL-1β, a key neuroinflammatory marker, was examined through inhibition of this pathway using a caspase-4 inhibitor and caspase-4-specific siRNA. *P. gingivalis*-LPS induced upregulation of caspase-4 expression, which was effectively suppressed using caspase-4-specific inhibitor Ac-LEVD-CHO confirming the specificity of the inhibitor’s action (Supplementary Figure S1). Notably, *P. gingivalis*-LPS transfection significantly upregulated IL-1β expression and this upregulation was reversed by treatment with the caspase-4 inhibitor Ac-LEVD-CHO (Figure 1A). Western blot analysis revealed the activation of the caspase-4-NLRP3-caspase-1-gasdermin D pathway, mediating IL-1β production in *P. gingivalis*-LPS-transfected SH-SY5Y cells (Figure 2B–H), and also in HMC3 cells (Supplementary Figure S2). We assessed the role of caspase-4 in the processing of caspase-1 and release of IL-1β in response to *P. gingivalis*-LPS by using caspase-4-specific siRNA to knock down caspase-4 expression in both SH-SY5Y and HMC3 cells. Silencing of caspase-4 caused a significant attenuation of caspase-4-dependent activation of the noncanonical inflammasome pathway and subsequent IL-1β secretion compared with the negative control scrambled siRNA (Figure 2B–H; Supplementary Figure S2). These findings demonstrate that caspase-4 plays a crucial role in the *P. gingivalis*-LPS-mediated activation of the noncanonical inflammasome pathway, leading to the upregulation of IL-1β production and that inhibition or silencing of caspase-4 significantly attenuates this inflammatory response.

### 3.2. P. gingivalis-LPS Activates the AD-Associated Presenilin and Amyloid Secretase Pathway Mediated by Caspase-4

To investigate the role of caspase-4 in the development of amyloid-related pathologies induced by *P. gingivalis*-LPS, we used SH-SY5Y cells due to the absence of detectable release of Aβ_1–42_ and Aβ_1–40_ peptides in *P. gingivalis*-LPS-transfected HMC3 microglial cells [31]. We utilized caspase-4-specific siRNA and the caspase-4 inhibitor Ac-LEVD-CHO to achieve targeted knockdown of caspase-4 in SH-SY5Y cells. Our results demonstrated that *P. gingivalis*-LPS transfection resulted in the activation of APP and PS1 in SH-SY5Y cells, with caspase-4 silencing significantly reducing the expression of these proteins compared to the scrambled siRNA (Figure 2A–C). PS1, a key γ-secretase component, is essential for amyloidogenic APP processing and generating Aβ_1–42_. Interestingly, *P. gingivalis*-LPS transfection significantly increased the release of Aβ_1–42_ and Aβ_1–40_ peptides relative to non-transfected control cells, and this upregulation was reversed by treatment with caspase-4 siRNA (Figure 2E,F) as well as with the caspase-4 inhibitor Ac-LEVD-CHO (Figure 2G,H). Furthermore, we also investigated the impact of caspase-4 on reelin expression, a glycoprotein that contributes to AD in association with Aβ. *P. gingivalis*-LPS transfection led to the upregulation of reelin protein expression in SH-SY5Y cells, and caspase-4 silencing significantly attenuated its expression compared to the scrambled siRNA (Figure 2A,D). Collectively, these findings provide strong evidence that caspase-4 mediates *P. gingivalis*-LPS-induced neuroinflammation and highlight the complex interactions between caspase-4, APP, PS1, and Aβ in the pathogenesis of AD and ADRD.

### 3.3. Caspase-4 Drives P. gingivalis-LPS-Induced Upregulation of Neuroinflammatory Markers

**Figure 3:**
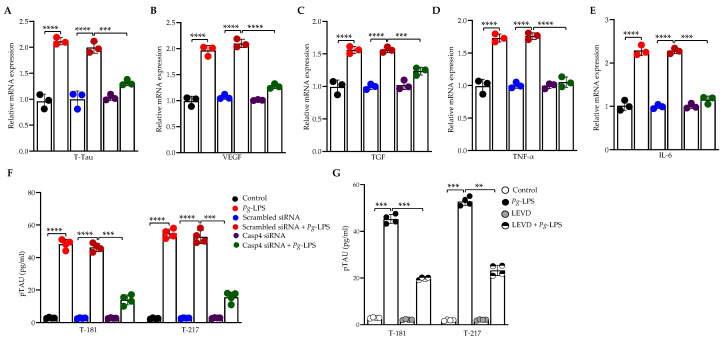
*P. gingivalis*-LPS induces neuroinflammatory markers via caspase-4 activation in SH-SY5Y cells. Relative mRNA expression of neuroinflammatory markers, including (**A**) T-Tau (Total Tau), (**B**) VEGF, (**C**) TGF-β, (**D**) TNF-α, and (**E**) IL-6, was significantly increased following *P. gingivalis*-LPS treatment and reversed by caspase-4 silencing using siRNA (*n* = 3). (**F**,**G**) ELISA analysis of phosphorylated tau at T181 and T217 in response to *P. gingivalis*-LPS treatment along with caspase-4 siRNA, and a caspase-4 inhibitor, Ac-LEVD-CHO (*n* = 4). Data are expressed as mean ± SEM and represent at least three independent experiments. Statistical significance is indicated as follows: ** *p* < 0.01; *** *p* < 0.001; **** *p* < 0.0001 determined by one-way ANOVA with Tukey’s multiple comparisons test.

Our previous studies have shown that *P. gingivalis*-LPS significantly activates neuroinflammatory and dementia markers [31,49]. To elucidate the role of caspase-4 in *P. gingivalis*-LPS-induced expression of neuroinflammatory proteins, we performed RT-qPCR of *P. gingivalis*-LPS-transfected SH-SY5Y cells, pretreated with caspase-4 siRNA, and *P. gingivalis*-LPS-transfected HMC3 cells, pretreated with caspase-4 inhibitor Ac-LEVD-CHO. The mRNA levels of total tau (t-tau), VEGF, TGF-β, TNF-α, and IL-6 were significantly upregulated in *P. gingivalis*-LPS-transfected SH-SY5Y and HMC3 cells compared to controls (Figure 3A–E; Supplementary Figures S2E and S3A). Notably, silencing or inhibition of caspase-4 markedly reduced the elevated expression of these neuroinflammatory markers relative to the negative controls (Figure 3A–E; Supplementary Figures S2E and S3A). Furthermore, we observed significant upregulation of p-tau at Thr181 and Thr217 in *P. gingivalis*-LPS-transfected SH-SY5Y cells, as measured by ELISA, compared to non-transfected controls (Figure 3F,G). However, this effect was not observed in HMC3 cells [49]. This upregulation of p-tau at Thr181 and Thr217 in *P. gingivalis*-LPS-transfected SH-SY5Y cells was reversed following treatment with caspase-4 siRNA (Figure 3F) and the caspase-4 inhibitor Ac-LEVD-CHO (Figure 3G). Collectively, these findings confirm that caspase-4 plays a critical role in *P. gingivalis*-LPS-induced neuroinflammation.

### 3.4. P. gingivalis-LPS Induces Oxidative Stress and Decreases Mitochondrial Membrane Potential Mediated by Caspase-4

**Figure 4:**
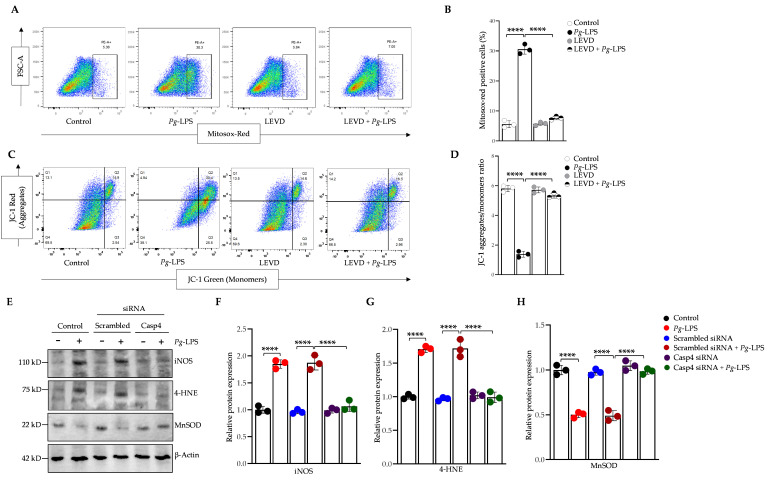
*P. gingivalis*-LPS induces oxidative stress and disrupts mitochondrial membrane potential via caspase-4 dependent inflammasome activation. SH-SY5Y cells were pretreated with Ac-LEVD-CHO for 1 h before LPS treatment. (**A**) ROS-producing cells were analyzed by flow cytometry using MitoSOX Red. (**B**) Bar graph showing a significant increase in the percentage of MitoSOX-positive cells upon LPS treatment, which was significantly reversed with Ac-LEVD-CHO. (**C**) Mitochondrial membrane potential was assessed using JC-1 staining. (**D**) The graph shows the ratio of JC-1 aggregates (red) to monomers (green), which was significantly decreased following LPS treatment and restored upon caspase-4 inhibition with Ac-LEVD-CHO. (**E**) Representative Western blots display the upregulation of iNOS and 4-HNE, along with the downregulation of MnSOD in response to *P. gingivalis*-LPS, with significant recovery of these markers following caspase-4 silencing via siRNA (*n* = 3). β-Actin was used as a loading control. (**F**–**H**) Quantification of relative protein expression of iNOS, 4-HNE, and MnSOD, normalized to β-Actin, is presented in the graphs (*n* = 3). Data are expressed as mean ± SEM and represent at least three independent experiments. Statistical significance is indicated as follows: **** *p* < 0.0001, determined by one-way ANOVA with Tukey’s multiple comparisons test.

In the context of oxidative stress induced by *P. gingivalis*-LPS-mediated ROS production, we investigated the potential role of caspase-4 in mitigating oxidative damage using the caspase-4 inhibitor Ac-LEVD-CHO and caspase-4-specific siRNA. ROS production within the mitochondria of live cells was selectively detected using MitoSOX Red, a fluorogenic dye specific for superoxide. MitoSOX-positive cells were subsequently isolated and analyzed by flow cytometry. Our results demonstrated that *P. gingivalis*-LPS transfection significantly increased ROS production in SH-SY5Y cells, an effect that was effectively inhibited by Ac-LEVD-CHO treatment (Figure 4A,B). Additionally, MMP, an indicator of mitochondrial function, was assessed using JC-1 dye. Aggregates of JC-1 in healthy mitochondria fluoresce red, whereas depolarized mitochondria result in JC-1 remaining in its monomeric form, which fluoresces green. Following *P. gingivalis*-LPS transfection, a significant decrease in JC-1 aggregates was observed, indicating mitochondrial depolarization and a reduction in ATP production, as evidenced by the shift from red to green fluorescence (Figure 4C,D). Notably, treatment with Ac-LEVD-CHO in the presence of *P. gingivalis*-LPS restored MMP, underscoring the protective role of caspase-4 inhibition in preserving mitochondrial function and cellular integrity (Figure 4C,D).

We further assessed iNOS protein expression, a crucial mediator in the synthesis of nitric oxide during oxidative stress, which plays a pivotal role in modulating inflammatory responses and contributing to cellular damage. Western blot analysis of iNOS revealed a significant upregulation following *P. gingivalis*-LPS transfection in SH-SY5Y cells. This upregulation was significantly reduced upon treatment with caspase-4 siRNA, restoring iNOS levels to baseline compared to control conditions (Figure 4E,F). Additionally, the oxidative stress biomarker 4-HNE was markedly elevated in *P. gingivalis*-LPS-transfected SH-SY5Y cells, but this increase was reversed with caspase-4 siRNA treatment (Figure 4E,G). Further, we assessed the expression of the antioxidant enzyme MnSOD, a critical component of the cellular defense against oxidative stress. Following *P. gingivalis*-LPS transfection, MnSOD expression was significantly reduced, suggesting a compromised antioxidant defense and a potential exacerbation of oxidative stress (Figure 4E,H). Notably, caspase-4 siRNA treatment restored MnSOD expression levels comparable to those of control cells, further indicating its role in the modulation of oxidative stress and cellular redox balance (Figure 4E,H). Collectively, our findings demonstrate that caspase-4 inhibition plays a critical protective role in mitigating oxidative stress and preserving mitochondrial function in *P. gingivalis*-LPS-induced cellular damage.

### 3.5. Caspase-4 Modulates Mitochondrial Biogenesis, Fission, and Fusion in Response to P. gingivalis-LPS

**Figure 5:**
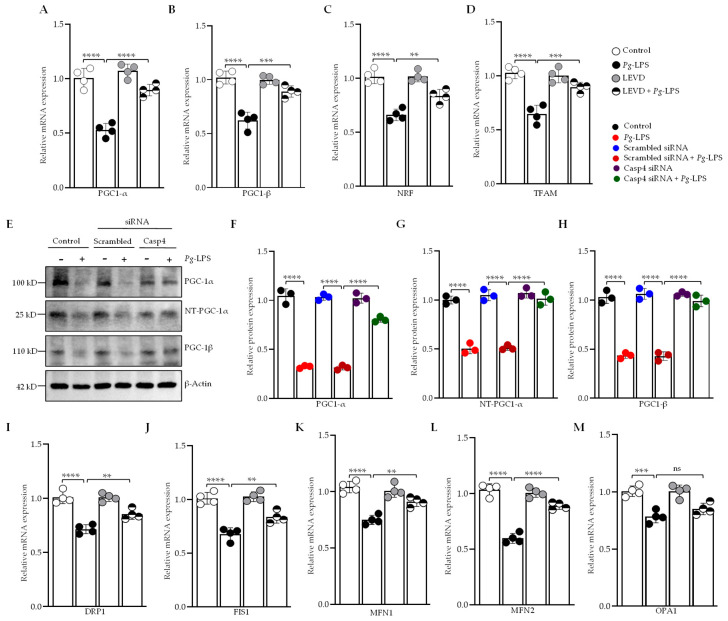
*P. gingivalis*-LPS inhibits mitochondrial biogenesis, fission, and fusion through caspase-4 activation. (**A**–**D**) RT-qPCR analysis of mitochondrial biogenesis markers, including PGC-1α, PGC-1β, NRF, and TFAM, revealed a significant downregulation of their mRNA expression following *P. gingivalis*-LPS treatment, which was significantly restored by caspase-4 inhibition using Ac-LEVD-CHO (*n* = 4). (**E**) Representative Western blots show the downregulation of PGC-1α, NT-PGC-1α, and PGC-1β in response to *P. gingivalis*-LPS, with recovery observed following caspase-4 silencing via siRNA. (**F**–**H**) Quantification of relative protein expression of PGC-1α, NT-PGC-1α, and PGC-1β, normalized to β-Actin, is presented in the graphs (*n* = 3). (**I,J**) RT-qPCR analysis of mitochondrial pro-fission (Fis1, Drp1) and (**K**–**M**) pro-fusion (Mfn1, Mfn2, Opa1) markers showed a significant decrease in their mRNA expression following LPS treatment, which was rescued by Ac-LEVD-CHO (*n* = 4). Data are expressed as mean ± SEM and represent at least three independent experiments. Statistical significance is indicated as follows: ** *p* < 0.01; *** *p* < 0.001; **** *p* < 0.0001; ns: *p* > 0.05, determined by one-way ANOVA with Tukey’s multiple comparisons test.

Mitochondrial biogenesis is regulated by the expression of mitochondrial-encoded genes, a process orchestrated by key transcriptional regulators. We investigated the role of caspase-4-dependent modulation of mitochondrial biogenesis in response to *P. gingivalis*-LPS by performing RT-qPCR analysis on *P. gingivalis*-LPS-transfected SH-SY5Y and HMC3 cells pretreated with Ac-LEVD-CHO or caspase-4 siRNA. Our results revealed a significant downregulation in the expression of *PGC-1α, PGC-1β, NRF1*, and *TFAM* mRNA in both *P. gingivalis*-LPS-transfected SH-SY5Y and HMC3 cells (Figure 5A–D; Supplementary Figure S4A–D). A significant decrease in the protein levels of PGC-1α, its splice variant NT-PGC-1α, and isoform PGC-1β was also observed in *P. gingivalis*-LPS-transfected SH-SY5Y cells (Figure 5E–H). Notably, this downregulation was significantly rescued by treatment with caspase-4 siRNA or Ac-LEVD-CHO (Figure 5A,C; Supplementary Figure S4A–D).

Additionally, *P. gingivalis*-LPS transfection led to alterations in genes involved in mitochondrial fission and fusion. The mRNA levels of mitochondrial fission-related genes, including dynamin-related protein 1 (*Drp1*) and mitochondrial fission 1 (*Fis1*), were significantly reduced (Figure 5I,J; Supplementary Figure S4E,F), along with marked downregulation of mitochondrial fusion genes such as mitofusin-1 (*Mfn1*), mitofusin-2 (*Mfn2*), and optic atrophy 1 (*Opa1*), which regulate fusion of the inner mitochondrial membrane (Figure 5K–M; Supplementary Figure S4G–I). The changes in fission and fusion gene expression were significantly reversed following treatment with Ac-LEVD-CHO, with the exception of *Opa1* in SH-SY5Y cells (Figure 5I–M; Supplementary Figure S4E–I). These results conclude that caspase-4 plays a key role in regulating mitochondrial biogenesis, fission, and fusion in response to *P. gingivalis*-LPS, with its inhibition significantly reversing these effects.

### 3.6. Caspase-4 Mediates the Impact of P. gingivalis-LPS on Oxidative Phosphorylation, Glycolysis, and ATP Production

**Figure 6:**
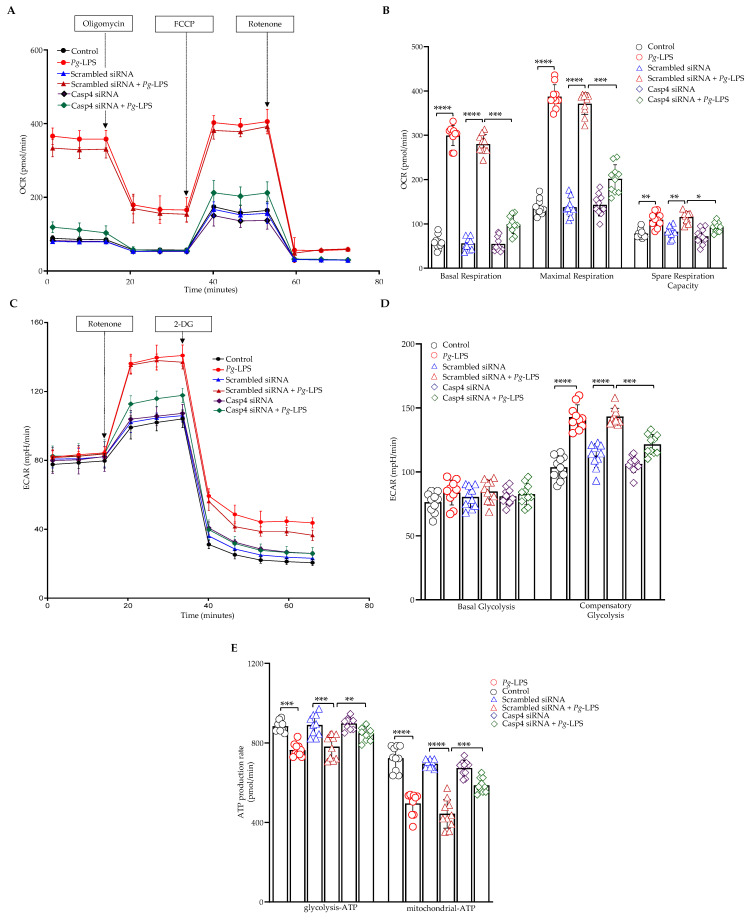
Mitochondrial functional analysis of OCR, ECAR, and total ATP production in SH-SY5Y cells. Cells were transfected with caspase-4 siRNA for 24 h before *P. gingivalis*-LPS treatment. (**A**) Seahorse XF Cell Mito Stress Test results showing mean ± SEM normalized to an equal number of cells. Oligomycin, FCCP, and rotenone were sequentially injected to assess mitochondrial ATP production, maximal respiration, and non-mitochondrial respiration. (**B**) Quantification of basal respiration, maximal respiration, and spare respiratory capacity revealed a significant increase following LPS treatment, which was reversed by caspase-4 siRNA. (**C**) Glycolytic rate assay data presented as mean ± SEM and normalized to equal cell numbers. Rotenone/antimycin A was injected to inhibit mitochondrial function, and 2-DG was used to block glycolysis as an internal control. (**D**) Graphs show the calculated glycolytic parameters, including basal and compensatory glycolysis. Compensatory glycolysis was significantly increased following LPS treatment and restored by caspase-4 siRNA. (**E**) Real-time ATP rate assay indicated a significant reduction in total ATP production from both oxidative phosphorylation and glycolysis following LPS treatment, which recovered upon caspase-4 silencing (*n* = 10). Data are expressed as mean ± SEM, representing at least three independent experiments. Statistical significance is indicated as follows: * *p* < 0.05; ** *p* < 0.01; *** *p* < 0.001; **** *p* < 0.0001, determined by two-way ANOVA with Tukey’s multiple comparisons test.

We investigated the role of caspase-4 in *P. gingivalis*-LPS-induced alterations in cellular energy metabolism by assessing two primary energy pathways, oxidative phosphorylation and glycolysis, along with their effects on ATP production using caspase-4 siRNA in SH-SY5Y cells. Our findings revealed that *P. gingivalis*-LPS transfection significantly altered mitochondrial function, as indicated by an increased OCR. This increase resulted in elevated basal respiration, maximal respiratory capacity, and spare respiratory capacity, which were all reversed upon caspase-4 siRNA treatment (Figure 6A,B). In the glycolytic pathway, basal glycolysis remained unaffected; however, compensatory glycolysis was enhanced following *P. gingivalis*-LPS treatment, with recovery observed upon caspase-4 siRNA treatment (Figure 6C,D). Furthermore, ATP levels both at basal conditions and after serial addition of mitochondrial inhibitors (oligomycin and rotenone/antimycin A) were reduced after *P. gingivalis*-LPS transfection but significantly improved following caspase-4 siRNA treatment. The data presented here demonstrated total cellular ATP production rates and pathway-specific mitoATP and glycoATP production rates in real time (Figure 6E). These results demonstrate that caspase-4 plays a crucial role in mediating *P. gingivalis*-LPS-induced alterations in cellular bioenergetics.

### 3.7. P. gingivalis-LPS Specifically Regulates Mitochondrial Respiratory Complexes Through Caspase-4

**Figure 7:**
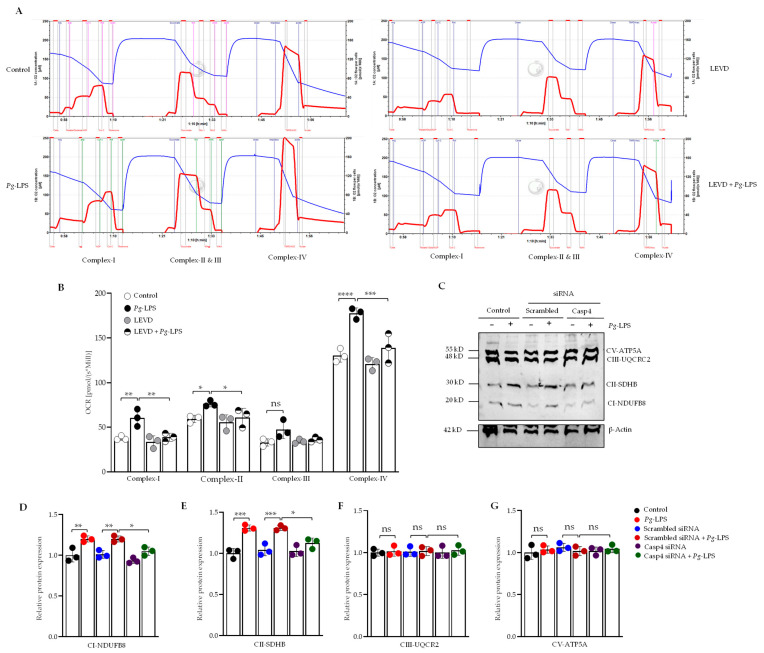
Functional analysis of mitochondrial respiratory chain activity in *P. gingivalis*-LPS transfected SH-SY5Y cells. Cells were treated with *P. gingivalis*-LPS with or without the caspase-4 inhibitor Ac-LEVD-CHO, then permeabilized with digitonin to assess the oxygen consumption rate (OCR) at various electron transport chain complexes (*n* = 3). OCR is shown as a function of time, with blue lines indicating the time points of substrate and inhibitor injections. (**A**) Representative trace from high-resolution respirometry using a multiple substrate-inhibitor titration protocol. The protocol includes malate/glutamate (complex I substrates), ADP (OXPHOS capacity), rotenone (complex I inhibition), succinate (complex II substrate), antimycin A (complex III inhibition), ascorbate/TMPD (complex IV), and sodium azide (complex IV inhibition). Oxygen concentration is depicted by blue lines, and respiration rate is represented by red lines. (**B**) Quantification of OCR at complexes I, II, and IV shows significantly increased OCR in *P. gingivalis*-LPS treated cells, which was reversed by Ac-LEVD-CHO treatment. (**C**) Representative Western blot analysis of OXPHOS mitochondrial complexes in SH-SY5Y cells treated with *P. gingivalis*-LPS, with or without caspase-4 siRNA using cocktail antibody against complexes I–V. (**D**–**G**) Relative quantification of protein levels of complex I, II, III, and V, normalized to β-Actin, shows a significant increase in complex I and II protein expression following *P. gingivalis*-LPS treatment, which was reversed by caspase-4 silencing. No significant change in complex III and complex V protein expression was observed between control and *P. gingivalis*-LPS treated groups. Data are expressed as mean ± SEM, representing at least three independent experiments. Statistical significance is indicated as follows: * *p* < 0.05; ** *p* < 0.01; *** *p* < 0.001; **** *p* < 0.0001; ns: *p* > 0.05, determined by two-way ANOVA with Tukey’s multiple comparisons test.

We conducted functional substrate-inhibitor titration experiments to examine the role of caspase-4 activation in the *P. gingivalis*-LPS-induced alterations of various complexes within the electron transport chain (ETC) using the caspase-4 inhibitor Ac-LEVD-CHO in SH-SY5Y cells. The ETC complexes investigated included complex I (NADH/ubiquinone oxidoreductase), complex II (succinate dehydrogenase), complex III (cytochrome c reductase), and complex IV (cytochrome c oxidase). Our results revealed that *P. gingivalis*-LPS significantly increased respiration rates in complexes I, II, and IV, while complex III remained unaffected. These findings suggest that *P. gingivalis*-LPS increases cellular oxygen consumption by enhancing mitochondrial respiration, indicative of cellular stress that potentially contributes to mitochondrial dysfunction. Importantly, these alterations were reversed upon treatment with Ac-LEVD-CHO (Figure 7A). A quantitative analysis of oxygen respiration rates across different complexes is provided in Figure 7B.

Furthermore, we assessed the impact of caspase-4 activation on the protein expression of ETC complexes (I–V) in response to *P. gingivalis*-LPS using caspase-4 siRNA. Western blot analysis using OXPHOS antibody revealed significant upregulation of complex I and complex II protein levels, while complexes III and V showed no significant changes between control and *P. gingivalis* LPS-treated groups (Figure 7C–G). The upregulation of complexes I and II was reversed upon treatment with caspase-4 siRNA, confirming the involvement of caspase-4 in the regulation of mitochondrial function following *P. gingivalis*-LPS exposure (Figure 7C–E). These findings suggest that caspase-4 activation plays a pivotal role in modulating mitochondrial respiration and the expression of electron transport chain complexes I and II in response to *P. gingivalis*-LPS, and with caspase-4 inhibition effectively reversing these disruptions.

## 4. Discussion

The present study highlights the essential role of caspase-4 in mediating neuroinflammatory responses to *P. gingivalis*-LPS, which contributes to key pathological features of AD and ADRD. SH-SY5Y cells are widely utilized as a neuronal model due to their expression of multiple dopaminergic neuronal markers in both undifferentiated and differentiated states, as well as their retention of key biochemical and functional characteristics of neurons. In addition to neuronal SH-SY5Y cells, we utilized HMC3 cells, to more accurately replicate the neuroinflammatory responses underlying *P. gingivalis*-LPS-induced inflammation. Microglia serve as the resident immune cells of the brain and play a central role in maintaining neuronal homeostasis and mediating immune responses, making them a relevant model for studying microglia-mediated neuroinflammation. Specifically, our data demonstrate that caspase-4 activation in SH-SY5Y and HMC3 cells, in response to *P. gingivalis*-LPS, triggers the caspase-4-NLRP3-caspase-1-gasdermin D inflammasome pathway, resulting in increased secretion of IL-1β, a hallmark of neuroinflammation. These findings align with recent studies that implicate caspase-4 mediated activation of the noncanonical inflammasome pathway in response to *P. gingivalis*-LPS [31,49,62]. Our findings demonstrate that silencing caspase-4 via siRNA significantly reduced IL-1β secretion, confirming its central role in *P. gingivalis*-LPS-induced neuroinflammatory cascades. These findings highlight the pivotal role of caspase-4-dependent noncanonical inflammasome activation and cytokine release in neurodegenerative conditions [63,64,65,66,67]. Furthermore, the inhibition of caspase-4 with Ac-LEVD-CHO also reversed IL-1β upregulation, suggesting that targeting caspase-4 could mitigate neuroinflammation associated with AD and related disorders.

IL-1β not only promotes inflammation but also stimulates the production of APP and the activity of enzymes involved in Aβ generation, both of which are implicated in AD and ADRD pathology [68,69]. Our findings demonstrate that caspase-4 activation in response to *P. gingivalis*-LPS induces an upregulation of APP and PS1 expression in SH-SY5Y cells, leading to enhanced production of amyloid-beta peptides, specifically Aβ_1–42_ and Aβ_1–40_. In contrast, no detectable release of Aβ_1–42_ and Aβ_1–40_ peptides was observed in *P. gingivalis*-LPS-transfected HMC3 microglial cells [31]. Caspase-4 directly interacts with PS1 through its caspase recruitment domain, influencing APP processing and subsequent Aβ formation [70]. Inhibition of caspase-4 significantly reduced Aβ production, suggesting a novel mechanism by which *P. gingivalis*-LPS promotes amyloidogenesis. These findings further support the hypothesis that chronic periodontitis, characterized by systemic inflammation, could accelerate AD progression. Furthermore, silencing caspase-4 diminished both APP and PS1 expression, as well as Aβ secretion, confirming its role in amyloidogenic processing. These results are consistent with recent literature highlighting caspase-4 as a key mediator in modulating amyloidogenesis and neuroinflammation in AD [63,64,65,66]. Additionally, the upregulation of reelin, an AD-associated glycoprotein, in response to *P. gingivalis*-LPS and its restoration upon caspase-4 inhibition suggests an intricate interaction between caspase-4, amyloidogenesis, and AD-related proteins. Alterations in reelin expression, which forms amyloid deposits in AD, may contribute to cognitive decline by impairing neuronal plasticity [71,72,73]. This interaction between caspase-4, amyloidogenesis, and reelin expression may offer new insights into the mechanisms by which *P. gingivalis*-LPS contributes to neurodegeneration in AD and ADRD.

Our data further support the involvement of caspase-4 in the activation of various neuroinflammatory markers, including t-tau, VEGF, TGF-β, TNF-α, and IL-6, in response to *P. gingivalis*-LPS in both SH-SY5Y and HMC3 cells, all of which are associated with neurodegenerative diseases [31,49]. Silencing of caspase-4 notably attenuated the elevated expression of these markers, indicating that caspase-4 is integral to the inflammatory response induced by *P. gingivalis*-LPS. This observation aligns with findings that caspase-4 mediates the release of proinflammatory cytokines via the noncanonical inflammasome pathway [74,75,76]. Additionally, in SH-SY5Y cells, tau hyperphosphorylation at residues Thr181 and Thr217 was observed, an effect that was reversed upon inhibition of caspase-4. These results reinforce the hypothesis that caspase-4 activation contributes significantly to tau-related pathology, a hallmark of AD associated with neurodegeneration and cognitive decline [77,78,79]. Previous research has established that microglia maintain brain homeostasis under normal conditions, but their activation can trigger pro-inflammatory cytokine release that affects neuronal cells [31]. Moreover, *P. gingivalis*-LPS has also been shown to activate microglia and induce neuronal degeneration, while also promoting amyloidogenesis and tau pathology, which are pathological features associated with the accelerated progression of AD/ADRD [31].

Recent studies have identified mitochondria as essential regulators of neuroinflammation, with caspase-4 emerging as a key mediator of mitochondrial permeability transition-induced pyroptosis, thereby linking mitochondrial dysfunction to inflammatory cell death pathways [80,81]. The mitochondrial dysfunction observed upon *P. gingivalis*-LPS exposure, as indicated by increased ROS production and decreased MMP, was effectively reversed by caspase-4 inhibition, suggesting that caspase-4 mediates mitochondrial oxidative stress. This observation is consistent with previous studies indicating that *P. gingivalis* infection increases mtROS levels and decreases MMP, effects that are rescued by mitochondrial inhibitors such as Mdivi-1 [82]. The upregulation of iNOS and 4-HNE, oxidative stress markers, further implicates caspase-4 in exacerbating inflammation and cellular damage. Restoration of MnSOD expression upon caspase-4 inhibition underscores its role in antioxidant defense [57], further emphasizing the protective effects of caspase-4 inhibition against oxidative stress. Mitochondrial dysfunction is a well-established feature of neurodegenerative diseases, and our findings suggest that caspase-4 modulates mitochondrial dynamics in response to *P. gingivalis*-LPS in both SH-SY5Y and HMC3 cells. We observed significant downregulation of genes involved in mitochondrial biogenesis, such as *PGC-1α, PGC-1β, NRF1*, and *TFAM*, which aligns with our previous studies showing that *P. gingivalis*-LPS disrupts mitochondrial function [31,49]. These changes were reversed by caspase-4 inhibition, indicating that caspase-4 plays a crucial role in regulating mitochondrial biogenesis. Additionally, caspase-4 inhibition also reversed the disruption of mitochondrial fission and fusion gene expression, including *Drp1*, *Fis1*, *Mfn1*, and *Mfn2*, further supporting the role of caspase-4 in mitochondrial dynamics.

Our results provide strong evidence for the essential role of caspase-4 in mediating the alterations in cellular energy metabolism induced by *P. gingivalis*-LPS. Specifically, we observed a marked increase in mitochondrial function, as reflected by elevated OCR following *P. gingivalis*-LPS exposure, consistent with prior studies [49]. Additionally, while basal glycolytic activity remained unchanged, we noted a significant enhancement of compensatory glycolysis following *P. gingivalis*-LPS treatment. Furthermore, ATP production, derived from both OCR and glycolysis (ECAR), was significantly diminished after *P. gingivalis*-LPS exposure but restored upon caspase-4 siRNA treatment. Our findings suggest that *P. gingivalis*-LPS treatment significantly increased both mitochondrial respiration and glycolytic activity, likely due to oxidative stress driving a metabolic imbalance favoring ROS production over ATP synthesis, thereby contributing to neuroinflammatory processes. Consistent with our findings, a recent study also reported a decrease in total ATP levels, accompanied by a shift in cellular energy metabolism—likely resulting from impaired oxidative phosphorylation and a compensatory increase in glycolytic activity [83]. Interestingly, our findings highlight the crucial role of caspase-4 in maintaining cellular energy homeostasis during *P. gingivalis*-LPS-induced neuroinflammation. Furthermore, we examined the activity and expression of the ETC complexes to gain insights into how mitochondrial function is altered in response to stress induced by *P. gingivalis*-LPS. The oxygen consumption in complexes I, II, and IV of the ETC was increased, with no effect observed on complex III consistent with our previous study [49]. Others have demonstrated that incubation of gingival fibroblasts with *P. gingivalis*-LPS led to significantly elevated respiration rates at each phase of the respiration process, accompanied by a greater than threefold increase in complex IV protein expression [84].

Collectively, our observations of *P. gingivalis*-LPS induced increased respiratory activity and elevated oxygen consumption, along with a reduction in MMP, can be explained by a phenomenon called mitochondrial uncoupling where electron transport continues or is upregulated, but ATP synthesis becomes inefficient due to a compromised proton gradient across the inner mitochondrial membrane [84,85]. In our study, *P. gingivalis*-LPS likely induces oxidative stress that disrupts mitochondrial membrane integrity, which can increase proton leak or uncoupling protein activity, leading to elevated respiration rates as mitochondria attempt to compensate for reduced ATP output. At the same time, the loss of MMP reflects impaired membrane potential maintenance, further supporting a state of mitochondrial dysfunction rather than efficient energy production. Given that complexes I and II are major producers of ROS [40,41], our findings confirmed the upregulation of complexes I and II protein expression and suggest that these complexes may contribute significantly to ROS generation and inducing oxidative stress, which was reversed by caspase-4 inhibition, validating the involvement of caspase-4 in regulating mitochondrial function. Taken together, these findings underscore the critical role of caspase-4 in maintaining mitochondrial bioenergetics during inflammatory responses and highlight its potential as a therapeutic target in diseases characterized by disrupted mitochondrial homeostasis.

While our findings offer valuable insights into the role of caspase-4 activation in *P. gingivalis*-LPS infection, it is important to acknowledge potential limitations and the possibility of compensatory mechanisms that may influence the observed outcomes. Caspase-4 shares substantial structural and functional homology with other inflammatory caspases, particularly caspase-5 in humans and caspase-11 in mice, which may partially compensate for its loss or inhibition. Moreover, targeting caspase-4 could have broader effects on the inflammatory response, potentially modulating canonical inflammasome pathways or inducing alternative immune signaling cascades, depending on the specific cellular context and tissue environment. To fully elucidate the specificity and systemic impact of caspase-4 inhibition, further in vivo studies utilizing both pharmacological and genetic models will be essential.

## 5. Conclusions

Our findings provide robust evidence that caspase-4 is a pivotal mediator of neuroinflammation, amyloidogenesis, oxidative stress, and mitochondrial dysfunction in response to *P. gingivalis*-LPS. Targeting caspase-4 may offer a promising therapeutic strategy for mitigating the neuroinflammatory and mitochondrial disturbances induced by *P. gingivalis*-LPS. Future studies exploring the therapeutic potential of caspase-4 inhibition in animal models of AD and periodontitis will be valuable in advancing our understanding of its role in chronic inflammation and neurodegeneration associated with AD and ADRD.

## Data Availability

The raw data are available without reservation upon reasonable request.

## Supplementary Materials

The following supporting information can be downloaded at: https://www.mdpi.com/article/10.3390/cells14110804/s1, Figure S1: Representative Western blot depict the upregulation of caspase-4 in response to *P. gingivalis*-LPS, along with its suppression following caspase-4 inhibition using a caspase-4 inhibitor, Ac-LEVD-CHO. β-Actin was used as a loading control, Figure S2: *P. gingivalis*-LPS induces IL-1β secretion via a caspase-4–dependent non-canonical inflammasome pathway in HMC3 cells. Figure S3: *P. gingivalis*-LPS induces neuroinflammatory markers via caspase-4 activation in HMC3 cells. Figure S4: *P. gingivalis*-LPS inhibits mitochondrial biogenesis, fission, and fusion through caspase-4 activation in HMC3 cells. Table S1: List of primer sequences used for qPCR analysis.

## Abbreviations

The following abbreviations are used in this manuscript:

*P. gingivalis*	*Porphyromonas gingivalis*
LPS	Lipopolysaccharide
AD	Alzheimer’s disease
ADRD	Alzheimer’s disease and AD-related dementias
OMVs	Outer membrane vesicles
GSDMD	Gasdermin D
ROS	Reactive oxygen species
APP	Amyloid precursor protein
Aβ	Amyloid-β
PS1	Presenilin-1
iNOS	Inducible nitric oxide synthase
4-HNE	4-hydroxynonenal
MnSOD	Manganese superoxide dismutase
RT-qPCR	Real-time quantitative PCR
SDS-PAGE	Sodium dodecyl sulfate-polyacrylamide gel electrophoresis
TBST	Tris-buffered saline with Tween-20
mtROS	Mitochondrial reactive oxygen species
MMP	Mitochondrial membrane potential
OCR	Oxygen consumption rate
ECAR	Extracellular acidification rate
ATP	Adenosine triphosphate
ETC	Electron transport chain
OXPHOS	Oxidative phosphorylation
