# Supplementary material for: Porphyromonas gingivalis-Lipopolysaccharide Induced Caspase-4 Dependent Noncanonical Inflammasome Activation Drives Alzheimer’s Disease Pathologies
Source: Cells. 2025 May 30;14(11):804. doi: 10.3390/cells14110804 (PMC12153746; doi:10.3390/cells14110804)
Supplement: Supplementary file 1 [file cells-14-00804-s001.zip › cells-3649699-supplementary.pdf]

cells

Article

Porphyromonas gingivalis-lipopolysaccharide induced caspase-4 dependent noncanonical inflammasome activation drives Alzheimer's disease pathologies

Ambika Verma[1], Gohar Azhar[1], Pankaj Patyal[1], Xiaomin Zhang[1] and Jeanne Y. Wei[1*]

Supplementary Data

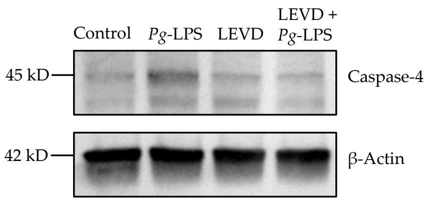

Supplementary Figure S1. Representative Western blot depict the upregulation of caspase-4 in response to *P. gingivalis*-LPS, along with its suppression following caspase-4 inhibition using a caspase-4 inhibitor, Ac-LEVD-CHO. β-Actin was used as a loading control.

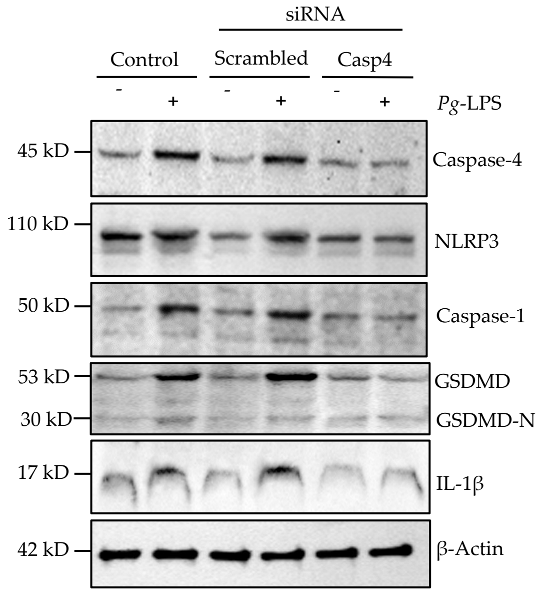

Supplementary Figure S2. *P. gingivalis*-LPS induces IL-1β secretion via a caspase-4–dependent non-canonical inflammasome pathway in HMC3 cells. Representative Western blots depict the upregulation of caspase-4, NLRP3, caspase-1, GSDMD, GSDMD-N and IL-1β in response to *P. gingivalis*-LPS, along with the reversal of this effect following caspase-4 silencing via siRNA. β-Actin was used as a loading control.

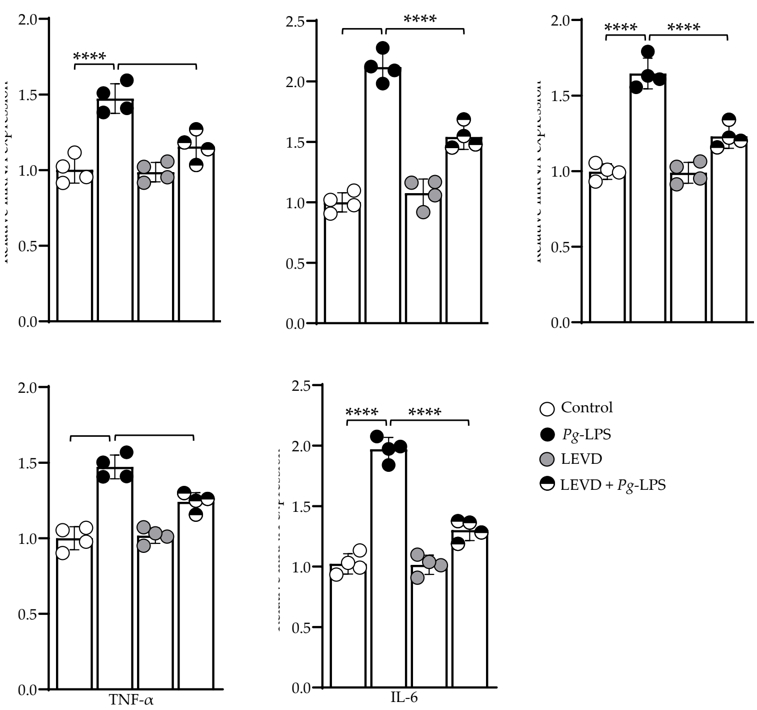

Supplementary Figure S3. *P. gingivalis*-LPS induces neuroinflammatory markers via caspase-4 activation in HMC3 cells. Relative mRNA expression of neuroinflammatory markers, including **(A)** T-Tau, **(B)** VEGF, **(C)** TGF-β, **(D)** TNF-α, and **(E)** IL-6, was significantly increased following *P. gingivalis*-LPS treatment and significantly reversed by caspase-4 inhibition using a caspase-4 inhibitor, Ac-LEVD-CHO ($n = 4$). Data are expressed as mean ± SEM and represent at least three independent experiments. Statistical significance is indicated as follows: * $P < 0.05$; ** $P < 0.01$; *** $P < 0.001$; **** $P < 0.0001$; ns: $P > 0.05$, determined by one-way ANOVA with Tukey's multiple comparisons test.

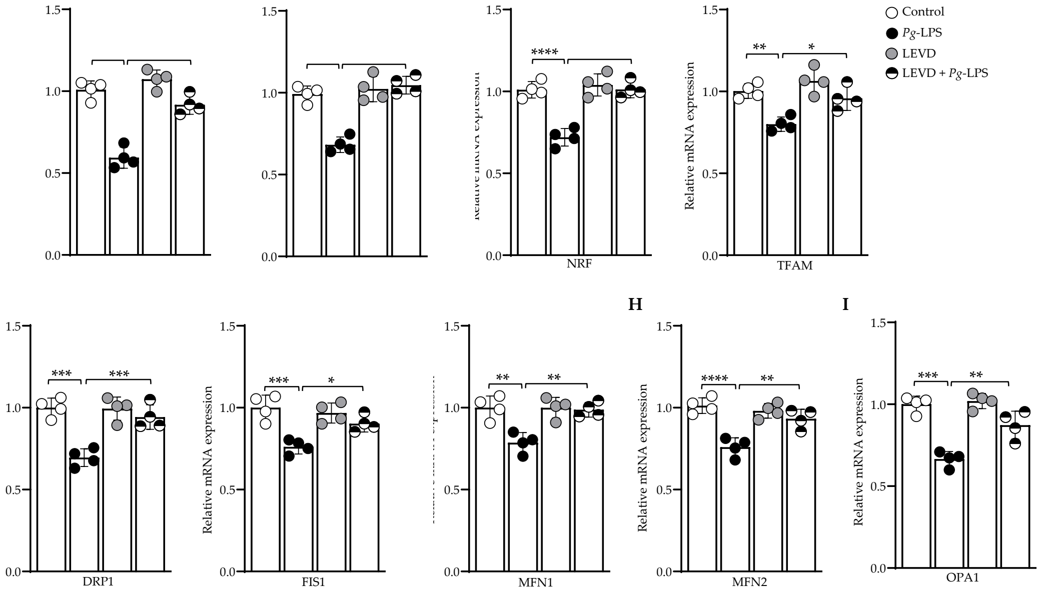

Supplementary Figure S4. *P. gingivalis*-LPS inhibits mitochondrial biogenesis, fission, and fusion through caspase-4 activation in HMC3 cells. **(A-D)** RT-qPCR analysis of mitochondrial biogenesis markers, including PGC-1α, PGC-1β, NRF and TFAM **(E, F)** RT-qPCR analysis of mitochondrial pro-fission (Fis1, Drp1) and **(G-I)** pro-fusion (Mfn1, Mfn2, Opa1) markers, which showed a significant decrease in their mRNA expression following LPS treatment, which was rescued by Ac-LEVD-CHO ($n = 4$). Data are

expressed as mean ± SEM and represent at least three independent experiments. Statistical significance is indicated as follows: * $P < 0.05$; ** $P < 0.01$; *** $P < 0.001$; **** $P < 0.0001$; ns: $P > 0.05$, determined by one-way ANOVA with Tukey's multiple comparisons test.

Supplementary Table S1. List of primer sequences used for qPCR analysis.

Primer Name	Primer Sequence (5'--3')
Tau-F	5′-GATTGGGTCCCTGGACAATA-3′
Tau-R	5′-GTGGTCTGTCTTGGCTTTGG-3′
VEGF-F	5′- TGCAGATTATGCGGATCAAAC C-3′
VEGF-R	5′-TGCATTCACATTTGTTGTGCTGTAC-3′
TGF-β-F	5′- GCGTGCTAATGGTGGAAACC-3′
TGF-β-R	5′- CGGAGCTCTGATGTGTTGAAGA-3′
TNF-α-F	5′-CAGTATGTGAGAGGAAGAGAACC-3′
TNF-α-R	5′-TCAGCAAGGACAGCAGAGG-3′
*IL-6*F	5′-ACTTGCCTGGTGAAAATCAT-3′
*IL-6*R	5′-CAGGAACTGGATCAGGACTT-3′
PGC-1α-F	5'- ACCCACAGAGAACAGAAACAG-3'
PGC-1α-R	5'- GGGTCAGAGGAAGAGATAAAGTTG-3'
PGC-1β –F	5'- ACTACTTCGCTGACACGCAG-3'
PGC-1β –R	5'- CTCTGAGTTCTCTGGGCACC-3'
NRF1-F	5'- GGAAACGGCCTCATGTATTTG-3'
NRF1-R	5'- GTTTGGAGGGTGAGATACAGAG-3'
TFam-F	5'- TTTCTCCGAAGCATGTGGG-3'
TFam-R	5'-GCCAAGACAGATGAAAACCAC -3'
DRP1-F	5′-GGTGAACCCGTGGATGATAAA-3′
DRP1-R	5′-CCTCAGGCACAAATAAAGCAG-3′
MFN1-F	5'- TGGCATCTGTGGCCGAGTT-3'
MFN1-R	5'-GAAACAGGTTCTGCCATTATGCT -3'
MFN2-F	5'- CGCGCTTATCCACTTCCCTC-3'
MFN2-R	5'- AGAAGAGCAGGGACATTGCG-3'
Fis1-F	5'- TGACATCCGTAAAGGCATCG-3'
Fis1-R	5'-CTTCTCGTATTCCTTGAGCCG -3'
Opa1-F	5'- GGAGAACCATATTCGTTTTGACC-3'
Opa1-R	5'- AGAGCTGTTCCCTTTTCCTG-3'

F: forward primer; R: reverse primer.
